# Role of Epigallocatechin Gallate in Selected Malignant Neoplasms in Women

**DOI:** 10.3390/nu17020212

**Published:** 2025-01-08

**Authors:** Anna Markowska, Michał Antoszczak, Janina Markowska, Adam Huczyński

**Affiliations:** 1 Department of Perinatology and Women’s Health, Poznań University of Medical Sciences, 60-535 Poznań, Poland; annamarkowska@vp.pl; 2 Department of Medical Chemistry, Faculty of Chemistry, Adam Mickiewicz University, 61-614 Poznań, Poland; michant@amu.edu.pl; 3 Gynecological Oncology Center, Poznańska 58A, 60-850 Poznań, Poland

**Keywords:** natural polyphenols, gynecological cancers, in vitro, in vivo, synergistic effects, drug delivery systems, green tea

## Abstract

Tea is a significant source of flavonoids in the diet. Due to different production processes, the amount of bioactive compounds in unfermented (green) and (semi-)fermented tea differs. Importantly, green tea has a similar composition of phenolic compounds to fresh, unprocessed tea leaves. It consists primarily of monomeric flavan-3-ols, known as catechins, of which epigallocatechin gallate (EGCG) is the most abundant. Thanks to its antioxidant, antiproliferative, and antiangiogenic properties, EGCG has attracted the scientific community’s attention to its potential use in preventing and/or combating cancer. In this review article, we summarize the literature reports found in the Google Scholar and PubMed databases on the anticancer effect of EGCG on selected malignant neoplasms in women, i.e., breast, cervical, endometrial, and ovarian cancers, which have been published over the last two decades. It needs to be emphasized that EGCG concentrations reported as effective against cancer cells are typically higher than those found in plasma after polyphenol administration. Moreover, the low bioavailability and absorption of EGCG appear to be the main reasons for the differences in the effects between in vitro and in vivo studies. In this context, we also decided to look at possible solutions to these problems, consisting of combining the polyphenol with other bioactive components or using nanotechnology. Despite the promising results of the studies conducted so far, mainly in vitro and on animal models, there is no doubt that further, broad-based activities are necessary to unequivocally assess the potential use of EGCG in oncological treatment to combat cancer in women.

## 1. Introduction

Cancer is a significant health issue in modern societies. Global statistics indicate that from 2020 to 2022, approximately 20 million people were diagnosed with cancer each year, and nearly 10 million cancer patients died annually from the disease [1,2]. The outlook for the coming decades is not encouraging. It is estimated that in 2040, over 28 million people will be diagnosed with cancer, representing a 47% increase compared to current statistics [1]. In 2050, this number is expected to rise to more than 35 million cases, indicating a 77% increase based on currently available data [2]. The increase in cancer-related deaths is projected to be even higher; Australian scientists estimate that by 2050, 8.8 million (90%) more people will die of cancer compared to 2022 [3].

There are several reasons for this upward trend, but one of the most significant is the anticipated demographic changes. In 2022, the global population was approximately 8 billion, and it is expected to exceed 9 billion by 2050 [2]. Cancer is a health and significant social problem due to its economic consequences. The loss of human potential, a notable reduction in productivity (presenteeism), long-term or complete exclusion from the labor market, the costs associated with treatment and care, and diminished social well-being are just a few of the adverse effects on the economy. These factors can ultimately lead to a deterioration of public finances, potentially threatening their stability.

Despite the substantial advancements in cancer treatment, the effectiveness of therapies for patients with cancer, particularly those with malignant tumors, remains inadequate. As a result, there is increasing interest in exploring potential strategies to enhance traditional cancer therapies, such as adjuvant treatments, that utilize specific natural products.

**Figure 1:**
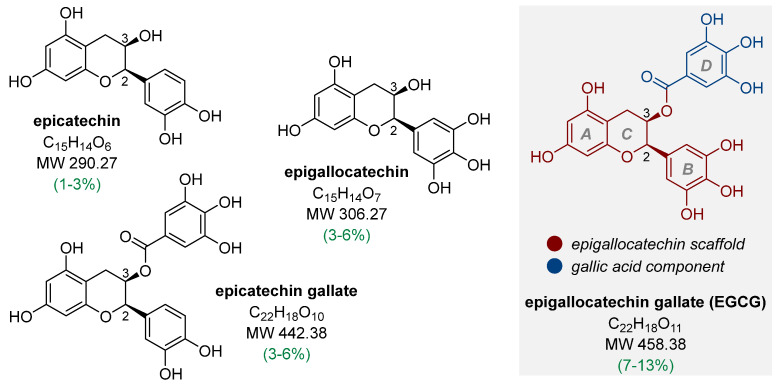
The structure of the main components of green tea from the group of natural polyphenols and their content in tea leaves (% of the dry mass of tea leaves, based on [7]).

Phenolic compounds are significant components of tea leaves, comprising 10–30% of the dry weight [4,5,6], but some literature sources indicate that this level can reach up to 36% [7,8]. The predominant compounds are catechins (flavan-3-ols), which account for up to approximately 30% of tea’s dry weight [9]. Variations in the tea chemical composition arise from such factors as plant cultivation conditions, geographical location, agricultural methods, and specific plant characteristics such as variety, age, and leaf position on the shoot [10]. Green tea, derived from the *Camellia sinensis* tea bush, is the second most popular beverage in the world after water, particularly in Asian countries. This unfermented tea contains various pharmacologically active compounds that have been successfully isolated and identified. These compounds offer a wide range of beneficial properties, including antioxidant, anti-inflammatory, hypoglycemic, antiviral, neuroprotective, cardioprotective, and anticancer effects [10,11,12,13,14,15,16]. In green tea production, heating the withered leaves deactivates endogenous oxidases, preventing the oxidation of the catechins. Fresh tea leaves and green tea share a similar composition of phenolic compounds [17]. There are four main types of monomeric catechins. Two of these catechins, (–)-epicatechin and (–)-epigallocatechin, have a free hydroxyl group at position C3. The other two are C3-esters of gallic acid, which are (–)-epicatechin gallate and (–)-epigallocatechin gallate (EGCG) (Figure 1). EGCG is regarded as the most intriguing compound in this group due to its abundant presence in green tea infusions, which can influence various physiological and pathological processes in humans [18].

The structure of EGCG consists of three aromatic rings (A, B, and D) and a heterocyclic ring, C, occupying the central position of the catechin molecule (Figure 1). Ring A, being the resorcinol component, is fused to ring C, while rings B (3,4,5-trihydroxyphenyl) and D (gallic acid residue) are connected to ring C directly or via an ester group at position C3 (Figure 1). The substituents at the C2 and C3 positions exhibit a *syn* (2*R*,3*R*) configuration, resulting in a (–)-epi(gallo)catechin composition (Figure 1). This differs from the (–)-(gallo)catechin arrangement with the corresponding substituents in an *anti* (2*S*,3*R*) configuration. The characteristic structure of EGCG, especially the presence of numerous hydroxyl groups, determines not only the favorable biological properties (including the highest antioxidant activity among catechins [19]) but also the relatively low bioavailability and metabolic stability of this natural polyphenol.

### 1.1. Bioavailability and Metabolic Biodegradation Pathways of EGCG

Although tea is a major source of dietary phenolic compounds, only a small portion can enter the bloodstream in their original form after consumption. The natural phenols in green tea travel through the mouth, esophagus, and stomach mostly unchanged. Only a limited percentage is absorbed by enterocytes and subsequently enters the circulatory system [17]. The pharmacokinetic properties of green tea polyphenols, especially EGCG, have been relatively well established in rodent studies (mice, rats), but there are some uncertainties about humans [20]. The total bioavailability of EGCG after oral administration of decaffeinated green tea to rats was only 0.1% [21]. In mice, polyphenol availability was higher (several percent) but still far from satisfactory [22]. In turn, studies conducted with the participation of healthy volunteers showed that after administering 800 mg of EGCG, the highest plasma concentration was 0.6–3.3 µM [23,24,25]. However, taking ≥800 mg of EGCG daily for 4 months or longer may be associated with increased alanine and aspartate aminotransferase levels, which may be a sign of liver dysfunction [26]. Nakagawa et al. [27] conducted a study involving four healthy individuals aged 21 to 28 years. They found that after the ingestion of 95 mg of polyphenol, the area under the plasma EGCG concentration-time curve was 857 ng·h/mL [27].

**Figure 2:**
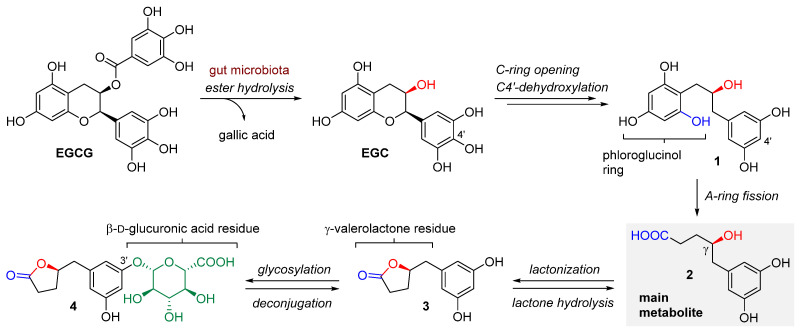
Proposed main metabolic pathways of EGCG degradation by gut microbiota (based on [32,33]), where EGCG = (–)-epigallocatechin gallate, EGC = (–)-epigallocatechin.

Limited bioavailability in the small intestine means that most phenols reach the large intestine in an unchanged form [28], raising questions about how these compounds can positively affect the body’s functioning. Native phenols found in green tea may possess health-promoting properties, at least in part, due to their direct impact on the microbiota, exhibiting prebiotic activity [17]. Additionally, some of these phenols can be converted into metabolites that are more accessible to the host by the microorganisms present in the large intestine (Figure 2) [29,30]. Specifically, the ester bond hydrolysis in EGCG, leading to gallic acid motif release, along with the C ring fission, is achieved exclusively by microbial enzymes, not mammalian ones [29]. Out of 169 strains of intestinal bacteria screened, *Enterobacter aerogenes*, *Raoultella planticola*, *Klebsiella pneumoniae* subsp. *pneumoniae*, and *Bifidobacterium longum* subsp. *infantis* have been identified as effective in hydrolyzing EGCG to EGC and gallic acid components, while *Eubacterium oxidoreducens*, *E. ramulus*, *E. casseli avus*, *Clostridium orbiscidens*, and the other ones belonging to the *Butyrivibrio* genus are involved in the fission of the C ring [29]. Different bacterial strains can also work together to degrade compounds from the catechin group [29]. Studies conducted on rats have demonstrated that when using EGCG labeled with tritium at position C4, the levels of radioactivity in the blood and urine were lower in the group that received initial antibiotic therapy compared to the control group that did not receive antibiotics [31]. Moreover, polyphenol absorption remained low for 4 h after oral administration, began to increase after 8 h, and reached maximum concentration 24 h post-ingestion [31].

The enzymatic activity of the intestinal microbiota enables efficient degradation of the organic compounds contained in tea, playing a key role in increasing the bioavailability of polyphenols, including EGCG. The principal metabolic pathway of EGCG degradation is based on the formation of (*R*)-5-(3,5-dihydroxyphenyl)-4-hydroxypentanoic acid **2** as the main metabolite through a series of transformations, including (i) hydrolysis of the ester group, (ii) opening of the C ring, (iii) removal of the hydroxyl group at position C4′, and (iv) cleavage of the A ring (Figure 2) [32,33]. The ɣ-hydroxyl acid metabolite **2** can, in turn, be converted into lactone **3** and then conjugated with glucuronic acid to form the 3′-*O*-β-glucuronide derivative **4** (Figure 2) [31,32,34]. Glucuronide conjugate **4**, which is classified as a phase II hepatic metabolite and reaches the colon via enterohepatic circulation, may undergo deconjugation and thus undergo further metabolism by the intestinal microbiota [35]. EGCG metabolites are generally less complex than the parent polyphenol and thus more readily absorbed in the large intestine [34].

### 1.2. Basic Epidemiological Data on Selected Malignant Neoplasms in Women

Breast cancer (BC) is the most common malignant tumor in women and the 2nd most common cause of cancer worldwide, regardless of gender [2]. In 2022, ~2.3 million women worldwide fell ill with this type of cancer, which constituted 11.6% of all recorded cancer cases, and ~666,000 patients died (6.9% of all cancer deaths) [2]. BC is a heterogeneous disease, both in terms of epidemiology (sporadic tumors, tumors with BRCA mutations) and the expression of receptors: estrogen (ER), progesterone (PR), and human epidermal growth factor receptor 2 (HER2). About 10–15% of all diagnosed BCs do not have these receptors; these are triple-negative BCs (TNBCs) with an aggressive course and poor prognosis [36]. These cancers are usually more common in premenopausal women (under 40 years of age) who are black and have a BRCA1 mutation [36].

Cervical cancer (CC) is one of the most common malignancies in women; it ranks 4th in terms of incidence among all female cancers [2]. In 2022, over 660,000 women were diagnosed with CC, representing 6.8% of all female malignancies [2]. Nearly 350,000 patients died from this disease, accounting for 8.1% of all deaths caused by female malignancies [2]. A detailed analysis of data on the mortality of oncological patients indicates that CC was the leading cause of cancer deaths in 37 out of 185 countries (20%) listed in the 2022 study [2]. Persistent infection with high-risk HPV viruses, which include 13 types, is closely associated with the development of dysplastic changes and CC; viral oncoproteins E6 and E7 are responsible for oncogenic activity [37]. The basic preventive strategy to protect against CC is vaccination against HPV, while in the case of cancer development, in addition to surgical treatment, cytotoxic chemical treatment is recommended.

Endometrial cancer (EC) is the 6th most commonly diagnosed malignant tumor in women in developed countries [2]. In 2022, more than 420,000 women were diagnosed with this type of cancer, which represented 4.3% of all malignant tumors in females, and nearly 98,000 patients died [2]. Unfortunately, the EC survival rate has decreased over the last four decades [38].

Ovarian cancer (OC) is the leading cause of death in women with cancer. In 2022, this type of cancer was diagnosed in over 320,000 women (3.4% of all cases of female cancer), and over 206,000 women died, which accounted for 4.8% of all cancer-related deaths among oncology patients [2]. Over 70% of this type of cancer is diagnosed in advanced stages; despite cytoreduction and chemotherapy, approximately 70–80% of patients experience relapses with an unfavorable course, and the cause of this phenomenon may be the presence of a small subpopulation of cancer stem cells (CSCs) inside cancer tumors [39,40,41]. Of note, phytochemicals can attack and destroy CSCs [42,43].

## 2. Anticancer Activity of EGCG

A growing body of research data suggests that EGCG may influence several fundamental biological processes and characteristics that contribute to the development and progression of cancer [44]. EGCG is a compound capable of attacking cancer cells [11,12,45] and CSCs [42]. The scientific literature describes in detail the mechanisms underlying the anticancer effects of EGCG, which include (i) inhibition of cancer cell proliferation and growth, (ii) inhibition of blood vessel formation (angiogenesis), (iii) inhibition of metastasis, (iv) induction of programmed cancer cell death (apoptosis), as well as (v) immunomodulatory activity through its influence on various signaling pathways [14,32,44,46,47,48,49,50,51]. Equally intriguing seems to be the possible synergistic effects of using EGCG in combination with known cytostatics, immunotherapeutic drugs, or other bioactive components [14,32,50,52,53,54]. Polychemotherapy based on combining EGCG with other substances may increase the polyphenol’s bioavailability and enhance the inhibition of cancer cell proliferation, induction of apoptosis, and suppression of angiogenesis or tumor growth [49,53].

The levels of EGCG found in plasma after administration may be significantly lower than the concentrations shown to be effective against cancer cells in in vitro tests (usually 10–100 µM) (Table 1). The efficient distribution of EGCG in the human body faces several natural barriers and processes [44,46]. Given the relatively low bioavailability of EGCG when taken orally due to factors such as low solubility in gastrointestinal fluids, slow intestinal absorption, limited permeability across the blood-brain barrier, and high susceptibility to degradation by intestinal microbiota [55], there is considerable interest in exploring the use of nanotechnology. Research in this area focuses on developing various systems, such as nanoparticles and nanoliposomes, that can effectively and selectively deliver the polyphenol in its native form to targeted sites. This approach may enhance the potential clinical applications of EGCG. Of particular note are nanosystems that could be successfully used in tests on BC cells and/or common gynecological cancers.

To the best of our knowledge, there has not been a recent review article summarizing the potential benefits of EGCG—whether used alone, in combination with other bioactive compounds, or enhanced through nanotechnology—in combating certain female cancers. Consequently, we searched the Google Scholar and PubMed databases for original studies published in the past 20 years that explored the effects of EGCG on breast, cervical, endometrial, and ovarian cancers (Table 1). We employed a variety of keyword combinations related to the polyphenol, such as “EGCG”, “epigallocatechin gallate”, or “epigallocatechin-3-gallate”, along with specific cancer types, including “breast cancer”, “cervical cancer”, “endometrial cancer”, as well as “ovarian cancer”. Furthermore, we reviewed the reference lists of the selected articles to identify additional relevant publications for inclusion in this review.

### 2.1. Breast Cancer

EGCG may modify estrogenic activity via ERα and ERα-dependent transcription [90]. Additionally, epidemiological studies indicate a possible association between culture-related green tea consumption and a lower risk of BC in some populations [66,91,92], suggesting potential clinical implications of EGCG in preventing and treating BC. Romano and Martel [93] summarized the broad spectrum of anticancer effects of EGCG based on the induction of apoptosis of BC cells by modulating the expression of various anti- and pro-apoptotic factors and angiogenesis inhibition. Concerning TNBC, EGCG inhibited the growth and proliferation of cancer cells by affecting estrogen receptor gene expression [94] or various cell signaling pathways and also increased cytostatic-induced apoptosis in these cells [95]. Moreover, reports on the effectiveness of synthetic analogs constructed based on EGCG [96,97,98], as well as the benefits of combining green tea and tamoxifen in the fight against BC in vitro and in vivo studies, seem to be interesting [99].

#### 2.1.1. Anti-Breast Cancer Activity of EGCG

A study published in 2017 found that EGCG inhibited the proliferation of MCF-7 cells in a concentration-dependent manner, with an IC_50_ value of 37.7 µM [56]. Additionally, EGCG promoted apoptosis, with an apoptosis index of 5.83% observed at a concentration of 30 µM (*p* = 0.0124) [56]. The authors associated these effects with polyphenol’s action on the p53/Bcl-2 pathway [56]. EGCG reduced telomerase activity (40–55% according to [57]), whose level is elevated in >90% of BC cases, thus becoming the subject of special attention in the context of diagnostic tests and the development of new anticancer therapies [57,58]. Polyphenol had no adverse effect on the growth of normal mammary gland cells [57]. Other studies have shown that EGCG can cause changes at the (epi)genetic level [59,100,101], suppress the activity of apoptosis inhibitors [102], affect steroid receptors [103,104], T-type calcium channels [105] and important signaling pathways [106,107,108], as well as inhibit cell growth, tumorigenesis, or invasiveness by modulating miRNA expression or activating MMPs [60,61,109,110]. Wei et al. [62] focused on the possible effect of polyphenol on glucose metabolism, which was inhibited by it, causing a reduction in the growth of BC cells in preclinical models. The activity of EGCG limiting glucose uptake by cancer cells was also confirmed by other authors [111]. In the studies conducted on mice that were given EGCG at doses of 50–100 mg/kg/day in their drinking water for four weeks, researchers observed its effects on angiogenesis and breast tumor growth [63]. The results showed a significant difference between the treatment group and the control group, with tumor growth measuring 0.37 ± 0.15 in the EGCG group compared to 1.16 ± 0.30 in the control group that did not receive EGCG (*p* < 0.01) [63]. The therapy was also associated with changes in the modulation of HIF-1α and NF-κB activation, as well as VEGF expression [63]; the effects on HIF-1α and VEGF were also observed in independent studies. [64]. Polyphenol did not negatively affect body weight, heart function, angiogenesis, or VEGF expression in the heart and skeletal muscle of mice [63].

Moreover, EGCG inhibited the proliferation, migration, and invasiveness of cells derived from stem-like inflammatory BC [65] or TNBC [67,68,69,112], among others, due to blocking of Wnt/β-catenin signaling in the latter group of tumors [68,112]. In vitro studies revealed the potential benefits of EGCG in inhibiting Met (hepatocyte growth factor receptor) signaling, which is a prognostic indicator of oncological treatment and survival of patients with BC [70]. Cancer development was delayed in the EGCG-treated mice inoculated with MDA-MB-231 human BC cells compared to the control group [66]. Immunohistochemical studies of tumor tissue sections also showed that EGCG inhibited proliferation and induced cell apoptosis [66].

On the other hand, other potentially beneficial effects of EGCG use during anticancer therapy include possible alleviation of post-radiation dermatitis symptoms. In a phase I clinical trial in a group of 24 patients with BC undergoing mastectomy who had dermatitis caused by adjuvant radiotherapy, the use of a polyphenol solution (40–660 µmol/L) sprayed onto the irradiation field resulted in a significant reduction of bothersome symptoms (redness, pain, burning, itching) [113]. Similarly, in a phase II double-blind, placebo-controlled clinical trial involving 180 patients with BC undergoing postoperative radiotherapy, prophylactic use of EGCG solution (660 µmol/L) reduced the severity and incidence of dermatitis [114]. The local application of EGCG effectively alleviated radiotherapy’s side effects in other clinical studies [115]. The molecular mechanisms underlying the beneficial effects of EGCG in acute radiation-induced skin toxicity are complex, involving antibacterial and anti-inflammatory processes [115].

#### 2.1.2. Combined Action of EGCG Against Breast Cancer

Using the MCF-7 BC cell model, Hsieh and Wu [116] tested whether the combination of EGCG, resveratrol, and γ-tocotrienol at suboptimal doses (10 µM) could induce enhanced inhibition of cell proliferation, modulate gene expression, or enhance antioxidant properties compared to the effects of each of the three phytochemicals administered alone. The synergy between the compounds was observed in the induction of quinone reductase NQO1, suggesting their possible beneficial cooperation [116]. EGCG, in combination with trastuzumab, may represent a novel strategy for the eradication of HER2-expressing BCs [117], and in combination with curcumin, increased cytotoxicity and intracellular levels of doxorubicin in drug-resistant MCF-7 cells [118]. Also, in studies on the MCF-7 cell line, EGCG increased the level of ERα, which made cancer cells more susceptible to the antagonist of this protein, tamoxifen [119]; other authors also confirmed the beneficial effect of the combination of EGCG and tamoxifen [120].

Importantly, Chisholm et al. [121] demonstrated synergistic cytotoxicity of EGCG combined with 4-hydroxytamoxifen (afimoxifene)—a selective ER modulator and an active metabolite of tamoxifen—on the MDA-MB-231 cell line. Another potential method of limiting the growth of highly invasive BC cells may be the administration of EGCG in combination with suberoylanilide hydroxamic acid (vorinostat), a histone deacetylase inhibitor associated with the inhibition of the transcription process [122,123]. The combined use of both compounds in vitro modulated, among others, the expression of cellular inhibitor of apoptosis 2 (cIAP2), proapoptotic caspase 7, oncogenic miRNA-221/222, and tumor suppressors p27 and PTEN, and finally reduced the metastatic potential and migration of cancer cells [122,123]. Studies on MDA-MB-231 cells showed that EGCG (25 µM) in combination with curcumin (3 µM) has a synergistic cytotoxic effect and promotes cell cycle arrest in the G2/M phase [124]. Combined treatment of mice with 25 mg/kg/day EGCG plus 200 mg/kg/day curcumin resulted in a 49% reduction in tumor volume compared to the control group (*p* < 0.05), which correlated with a 78 ± 6% decrease in VEGFR-1 protein expression level [124]. Both the proliferation and apoptosis of MDA-MB-231 cells were negatively affected by the administration of EGCG and tapentadol, an opioid analgesic, due to disrupted cell cycle progression (*p* < 0.05) [125]. EGCG has been identified as a compound that sensitizes tumor cells to paclitaxel, an oncological drug often used in BC treatment [126].

#### 2.1.3. EGCG-Based Nanosystems for Breast Cancer Treatment

Considering the limitations resulting from the relatively low solubility and bioavailability of EGCG (please see Section 1.1), worldwide research is underway on the possible use of nanotechnology to improve the anticancer properties of compounds belonging to the group of natural polyphenols [127]. Zeng et al. [128] constructed two types of EGCG-loaded nanoparticles to enhance the targeting of MCF-7 cells with polyphenol. The system developed by the authors not only improved this parameter but also increased the effectiveness of EGCG in inhibiting cancer cell proliferation (IC_50_ = 470.5 ± 33.0 μg/mL, IC_50_ = 65.9 ± 0.4 μg/mL and IC_50_ = 66.6 ± 0.6 μg/mL, respectively, for EGCG and two types of EGCG-containing nanoparticles modified with folic acid and polyethylene glycol) [128]. Moreover, EGCG-containing nanoparticles were found to be more active and more effective in inhibiting the development of aggressive forms of BC in mice overexpressing CCN5 than free polyphenols [129]. In a recent study, nanoparticles composed of EGCG, phosphatidylcholine, doxorubicin, and anthocyanin procyanidin significantly inhibited the growth of BT-474, EMT-6, MCF-7, and MDA-MB-231 cell lines [130]. EGCG, together with paclitaxel in the form of PLGA-casein core/shell nanoparticles or liposomal co-delivery system, effectively reduced the invasiveness of MDA-MB-231 cells [131,132].

In turn, EGCG-encapsulated chitosan-coated nanoliposomes increased cancer cells’ stability and intracellular polyphenol content, inhibiting their proliferation and inducing apoptosis [133]. This type of system maintained anticancer efficacy at a concentration of 10 µM or lower, at which EGCG in its native form was ineffective [133]. Similar results were obtained for EGCG encapsulated in lipid nanoparticles, whose cytotoxicity was over 8 times higher against MDA-MB-231 cells than that of free polyphenol [134]. Combining a similar system with bombesin, a peptide targeting gastrin-releasing peptide receptors overexpressed in BCs, resulted in increased specificity and improved cytotoxicity against cancer cell lines [135].

### 2.2. Cervical Cancer

More and more studies indicate the possible use of EGCG or its analogs in regulating HPV infection and thus preventing the occurrence of CC [71,136,137]. The use of a dietary supplement in a regimen of two tablets a day for eight weeks, containing EGCG (200 mg), vitamin B12 (1 mg), folic acid (400 μg), and hyaluronic acid (50 mg), effectively alleviated precancerous cytological changes of the cervix caused by HPV infection [138], which was confirmed by other authors in subsequent years [139]. Moreover, EGCG had a beneficial effect on treating anogenital warts caused by HPV [140,141]. EGCG can induce apoptosis of CC cells by stimulating the degradation of oncogenes/oncoproteins and modulating the expression of tumor suppressor genes, as well as cause the degradation of extracellular matrix components, including MMPs, which play a role in cancer invasion, migration, and metastasis [37].

#### 2.2.1. Anti-Cervical Cancer Activity of EGCG

The use of EGCG was associated with the degradation of E6 and E7 oncoproteins and increased expression of their associated tumor suppressor genes [142]. The limiting effect of EGCG on CC cell lines may be related to the modulation of miRNA expression [71] or fused toes homolog (FTS) mRNA via p53 [143]. Other potential mechanisms of the anticancer effect of EGCG may be the modulation of the angiogenesis signaling cascade [72] or the suppression of epithelial-mesenchymal transition (EMT) [73]. In studies on HPV-positive cancer cell lines (CaSki, HeLa, and SiHa), it was found that the addition of EGCG to cell culture inhibits their growth in a manner dependent on the concentration of polyphenol (*p* < 0.05) [71]. The limitation of CaSki (IC_50_ = 27.3 µM) and HeLa (IC_50_ = 47.9 µM) cell growth could result from cell cycle arrest and induction of apoptosis [74]. EGCG at a concentration of 5 µg/mL or 10 µg/mL increased the level of proapoptotic caspase 3 [144]. Other in vitro studies have shown that EGCG affects epigenetic modulators, signaling pathways, or suppressor genes [75,76]. The reduction in expression caused by EGCG concerned key signaling pathways (MAPK, PI3K, Wnt) regulating the cell cycle and the potential for metastasis of cancer cells [75]. Japanese researchers found that the anticancer mechanism of EGCG action may also be due to the inhibition of telomerase activity [77,78]. Moreover, a beneficial effect of EGCG on the inhibition of epidermal growth factor (EGF) activity has been described [79]. It has been established that elevated levels of EGF in the tumor microenvironment may be associated with the ability of tumor cells to metastasize; in cell culture of both HPV-positive and HPV-negative CC lines, EGCG effectively blocked the action of EGF [79].

#### 2.2.2. Combined Action of EGCG Against Cervical Cancer

**Figure 3:**
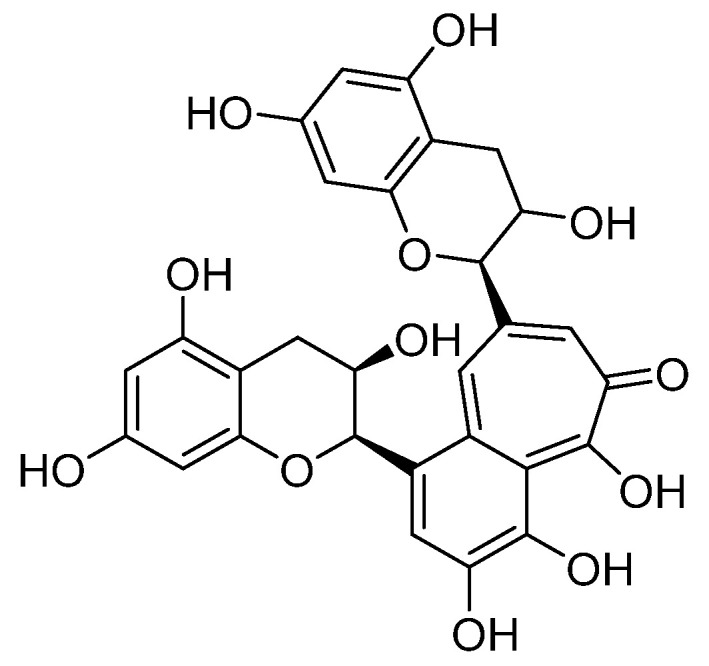
The structure of theaflavin, an important polyphenol found in black tea.

In the studies on the HeLa cell line, the anticancer effect of EGCG in combination with another polyphenolic compound present in black tea, theaflavin (Figure 3), was determined [145]. Both components acted synergistically, disrupting cancer cell growth due to microtubule depolymerization, which affected the reduction of PI3K/AKT pathway signaling and led to cell death by apoptosis [145]. An enhanced anticancer effect on HeLa cells was also demonstrated after using EGCG with eugenol and amarogentin [146]. In this case, the inhibitory effect on proliferation resulted from the decreased expression of cyclin D1 and the increased expression of cell cycle inhibitors [146]. Reports indicate that EGCG acts synergistically against cancer cell proliferation when combined with enoxacin, a fluoroquinolone agent showing antibacterial activity [147]. Similar beneficial effects were observed with EGCG (25 μM) and cisplatin (250 nM) [148]. EGCG and retinoic acid administered together induced apoptosis and inhibited telomerase activity in cancer cell lines, which was not observed for either of these natural compounds used alone [149]. On the other hand, according to Raish et al. [150], photodynamic therapy (PDT) combined with EGCG can induce a significant tumor suppressive response compared with PDT alone.

#### 2.2.3. EGCG-Based Nanosystems for Cervical Cancer Treatment

There are few literature reports on the influence of nanotechnology on the improvement of anti-CC properties of EGCG. For example, the use of gold nanoparticles containing paclitaxel and EGCG functionalized with poly-D-lysine grafted polyethylene glycol and triphenylphosphonium cations (TPP^+^) targeting mitochondria caused apoptotic death of HeLa cells used in the tests [151].

### 2.3. Endometrial Cancer

Interestingly, the results of some studies suggest that EGCG may have an impact on reducing the risk of occurrence and slowing the course of EC [80,152,153], including the genetic Lynch syndrome [154]. Huang et al. [50] summarized the potential of EGCG against EC cell lines, indicating, among others, the modulating effect of the polyphenol on selected signaling pathways and the nuclear transcription factor Nrf2, which resulted in inhibition of proliferation and invasive potential, as well as induction of apoptosis. Apart from EGCG in its native form, interesting results were also obtained using in vitro and in vivo tests with derivatives (prodrugs) obtained by chemical modification of the polyphenol molecule [155,156,157].

#### Anti-Endometrial Cancer Activity of EGCG

The studies performed by Manohar et al. [80] indicated that EGCG may limit EC cell proliferation by inhibiting ERK activation and inducing apoptosis mediated by reactive oxygen species (ROS) and activation of p38. The action of EGCG (100 µM) caused a decrease in the amount of VEGF isolated from the supernatant of cultured EC cells (82.39 ± 19.54 pg/mL vs. 436.06 ± 86.74 pg/mL in the control group, *p* < 0.0001), suggesting the potential of the polyphenol to inhibit the angiogenesis process [81].

### 2.4. Ovarian Cancer

A growing number of literature reports indicate a beneficial effect of EGCG on the course of OC [50,86,88,158,159,160,161]. The mechanism of the anticancer effect of EGCG in this type of cancer is diverse. It may include inhibition of proliferation and induction of cell apoptosis by modulating the expression of NF-κB, apoptotic proteins (Bax, Bcl-2), or caspase 3 [50]. Other described molecular mechanisms of EGCG action included disruption of the activation of protein kinases, reduction of VEGF production, and MMP activities [158].

#### 2.4.1. Anti-Ovarian Cancer Activity of EGCG

EGCG can inhibit the growth of OC cells and induce their apoptosis, including cisplatin-resistant cells [82], among others, by arresting the cell cycle, regulating proteins associated with it, or modulating p38 kinase and selected MMPs [83,84,85]. Polyphenols can also increase oxidative stress in cancer cells [162]. It has been proven that EGCG can exert a strong inhibitory effect on proliferation in tests on SKOV3 cells [86,87,163], which was accompanied by, among others, a decrease in the activity of the PTEN/AKT/mTOR pathway and an increase in the expression of the suppressor gene PTEN [86] or a reduction in the expression of AQP5 [163]. The effect on other important signaling pathways has also been described in the scientific literature [89]. The effect of EGCG on inhibiting OC growth has been observed in both in vitro and in vivo studies by various authors [88,89]. The molecular mechanism of action of EGCG consisted of the activation of the nuclear transcription factor FOXO3a responsible for cell hemostasis in response to changes in the extracellular and intracellular environment, including oxidative stress [88], and a decrease in basal and ET-1-induced angiogenesis and invasion tendency [89]. The use of EGCG also inhibited the expression of c-Myc, promoting the neoplastic process [88]. Moreover, EGCG administered to mice caused a stronger inhibitory effect on tumor growth compared to paclitaxel [86]. However, it should be noted that the dose of EGCG (50 mg/kg) was 10-fold higher than the dose of paclitaxel (5 mg/kg) [86].

In a single-arm phase II study of 16 patients with advanced stage III–IV OC who underwent cytoreductive surgery followed by chemotherapy, the effect of EGCG supplementation in maintenance therapy was investigated [160]. The study lasted 18 months, during which patients consumed 500 mL of EGCG-enriched green tea daily [160]. No disease relapses were observed in 5 of 16 women (~31%) enrolled in the study after 1.5 years of treatment [160]. At the same time, no toxicity was observed when drinking EGCG-enriched tea [160].

#### 2.4.2. Combined Action of EGCG Against Ovarian Cancer

EGCG and sulforaphane (an organosulfur compound from the isothiocyanate group with potential chemopreventive properties) induced apoptosis in paclitaxel-resistant OC cells by reducing the expression of hTERT and Bcl-2 [164]; the combination of these two compounds also enhanced cisplatin-mediated apoptosis [165]. Rodriguez Torres et al. [159] found that EGCG inhibited the growth of ovarian CSCs in a dose-dependent manner. Src expression was reduced, which increased the cytotoxicity of cisplatin and paclitaxel; a decrease in the activity of JAK/STAT3 signaling as well as the transcription factor NANOG was noted, which improved the efficacy of the mentioned oncological drugs [159]. EGCG increased sensitivity to cisplatin by regulating the expression of the copper and cisplatin influx transporter CTR1 [166] or by increasing oxidative stress [162] and also acted synergistically in combination with this chemotherapeutic [167]. The synergism of sequenced combinations of EGCG and curcumin with cisplatin in killing human OC cells has also been demonstrated [168].

In the clinical trial by Kiselev et al. [161], a method of potential maintenance treatment of advanced OC was described based on indole-3-carbinol (an indole derivative with multi-target anticancer activity) and EGCG, which was used in three of the five arms of the study together with chemotherapy (in the fourth arm only chemotherapy was applied, and in the fifth only observational treatment was performed). The study included 284 women with clinically advanced stage III–IV serous OC [161]. After five years, both progression-free survival (PFS) and overall survival (OS) were prolonged in women receiving additional EGCG treatment [161]. The median OS was 60 months in the arms of the study using the combination of indole-3-carbinol and EGCG compared to 46 months without these compounds [161]. Additionally, the percentage of patients with tumor relapse with ascites after combined treatment was also significantly lower [161].

#### 2.4.3. EGCG-Based Nanosystems for Ovarian Cancer Treatment

The nanoparticle-mediated systems can safely and target-specifically deliver various bioactive compounds, such as flavonoids, to tumors, improving their therapeutic potential [169,170,171]. The nanoparticles containing flavonoids can also enhance the effects of classical chemotherapy on cancer cells [171]. Several studies have already implemented the strategy of encapsulating EGCG for cancer treatment, including OC [172,173,174]. For example, to improve the targeting of cancer cells, Alizadeh et al. [175] prepared chitosan-coated silica nanoparticles and attached them to EGCG. The free amino groups of chitosan were then attached to the aptamer AS1411 [175]. The obtained nanomaterial improved the cytotoxic effect of EGCG (about 93% of cells underwent late apoptosis) [175]. Moreover, the functionalized nanoparticles caused a decrease in the number of cells in the S and G2/M phases, an increase in the G0/G1 phase, and cell arrest in the G1 phase [175]. A doxorubicin delivery system with folic acid-modified EGCG enhanced the uptake of an oncological drug by SKOV3 cells and effectively inhibited tumor growth in vivo [176]. On the other hand, Bae et al. [177] showed that micellar nanocomplexes with hyaluronic acid-EGCG conjugate and cisplatin enable efficient delivery of a platinum-based agent into CD44-overexpressing OC cells by receptor-mediated endocytosis (RME).

## 3. Conclusions

Epigallocatechin gallate (EGCG) is green tea’s primary polyphenol (catechin). EGCG has been widely studied for its potential health benefits, and its anticancer properties seem particularly interesting. Many authors have indicated polyphenol’s antiproliferative, antiangiogenic, and proapoptotic effects on various cancer cell lines and in vivo tests on animal models. EGCG can modulate signaling pathways associated with, among others, cell proliferation and division in multiple types of cancer, including breast cancer and gynecological cancers (cervical, endometrial, and ovarian). Despite the beneficial anticancer potential of EGCG, significant pharmacokinetic problems should be considered, which may affect the polyphenol’s ineffective systemic delivery and bioavailability. Various strategies have been adopted to improve the poor systemic bioavailability and cellular uptake of EGCG, including potential combination or polytherapy, which uses EGCG with one or more bioactive compounds. Furthermore, the approach using nanotechnology is fundamental, as it can help overcome the pharmacokinetic problems of EGCG by controlling its cytotoxicity and increasing bioavailability (the concept of nanochemoprevention).

This review article has been developed based on an analysis of data published over the last two decades. However, it should be noted that the topic of the potential beneficial effects of EGCG and other natural polyphenols has been so extensively explored that continuous updates will be necessary to keep up with emerging new research data. Given the encouraging anticancer activity of EGCG, it is important to validate and expand our understanding of the potential of this polyphenol against cancer cells. It should be made through both in vitro and in vivo tests, particularly using concentrations observed in plasma. The potential use of EGCG in chemopreventive treatment, adjuvant treatment, or supporting the action of classical oncological drugs also requires further verification, including clinical trials. Only on this basis will it be possible to draw more clear conclusions about the real anticancer potential of EGCG in the fight against selected malignant tumors in women.

## Figures and Tables

**Table 1:** Selected in vitro and in vivo studies on the anticancer potential of EGCG.

Cancer Type	Cancer Cell Lines	In Vitro EGCG Concentration	In Vivo Tests	Anticancer Effects	Ref.
Breast cancer	MCF-7	2, 4, 8, 16, 32, 64, 128 μM		Inhibition of proliferation, promotion of apoptosis	[56]
	MCF-7	10–100 μg/mL		Inhibition of reproductive potential and colony formation, reduction in cell viability, promotion of apoptosis	[57]
	T47D	10–80 μM		Reduction in cell viability, modulation of gene expression	[58]
	MCF-7, MDA-MB 231	20 μM		Inhibition of the progression of cancer	[59]
	MCF-7	0.5–20 μg/mL	CB-17 SCID mice (6–8 weeks old); EGCG dose: 100 mg/kg, oral gavage	Inhibition of cell viability, induction of apoptosis, disruption of the cell cycle in the G2/M phase, inhibition of tumor growth	[60]
	MCF-7	20 μM		Reduction in MMP-2 activity	[61]
	4T1	10–320 μM	BALB/c mice (4 weeks old); EGCG dose: 5, 10, 20 mg/kg, intraperitoneal injections	Reduction in cell growth, induction of apoptosis, promotion of mitochondrial depolarization, inhibition of glucose metabolism, reduction in tumor mass	[62]
	E0771, MCF-7, MDA-MB-231	10, 50 μg/mL	C57BL/6 mice (7 weeks old); EGCG dose: 50–100 mg/kg, drinking water	Impact on cancer cells and tumor blood vessels, reduction in tumor mass	[63]
	MCF-7	25, 50, 100 mg/L		Reduction in cell proliferation and growth	[64]
	SUM-149, SUM-190	5, 10, 20, 40, 60, 80, 160 µg/mL	NOD/SCID mice (6 weeks old); EGCG dose: 16.5 mg/kg, intraperitoneal injections	Reduction in cell growth, invasiveness, and survival, reduction in the growth of existing tumors	[65]
	MDA-MB-231	1–200 μg/mL	NCr-nu/nu mice (5 weeks old); EGCG dose: 1 mg in 100 μL of autoclaved distilled water	Inhibition of cell invasiveness, induction of apoptosis, delay in the occurrence of cancer, reduction in tumor mass	[66]
	Hs578T	10 μM		Inhibition of proliferation, migration, and invasiveness	[67]
	MDA-MB-231	0.5 μg/mL		Inhibition of invasion	[68]
	MDA-MB-231	50, 80 μg/mL		Inhibition of invasiveness, induction of apoptosis	[69]
	MDA-MB-231	0.07–20 μM		Inhibition of Met signaling, blocking invasiveness	[70]
Cervical cancer	C33A, CaSki, HeLa, SiHa	10, 20, 40, 60, 80, 100 µg/mL		Inhibition of cell growth, probably due to regulation of miRNA expression	[71]
	HeLa	10 μM		Inhibition of proliferation, adhesion, spread and invasiveness	[72]
	HeLa, SiHa	20, 40, 60, 80, 100 μM		Inhibition of cell viability	[73]
	CaSki, HeLa	10, 25, 50, 100 µM		Cell cycle arrest, apoptosis induction	[74]
	HeLa	50 µM		Modulation of epigenetic factors and signaling pathways (MAPK, PI3K, Wnt)	[75]
	HeLa	1–100 μM		Inhibition of cell growth, invasion, and migration	[76]
	OMC-4, TMCC-1	50–100 μM		Induction of apoptosis, inhibition of telomerase activity, dysregulation of the cell cycle	[77]
	HeLa, ME180, SiHa, TMCC-1	50, 100 μM		Induction of apoptosis, inhibition of telomerase activity	[78]
	C33A, ME180	50 μM		Reduction in matrix abundance and cell migration	[79]
Endometrial cancer	Ishikawa	50, 75, 100, 125, 150 μM		Inhibition of cell proliferation, induction of apoptosis, modulation of pro- and anti-apoptotic proteins, generation of ROS	[80]
	Ishikawa, RL952	100 μM		Reduction in VEGF	[81]
Ovarian cancer	A2780/DDP, SKOV3/DDP	0–40 μM	BALB/c mice (5 weeks old); EGCG dose: 50 mg/kg, intraperitoneal injections	Inhibition of cell proliferation and motility, induction of apoptosis, inhibition of tumor growth	[82]
	OVCAR-3, PA-1, SKOV-3	10–100 μM		Inhibition of cell growth, cell cycle arrest, induction of apoptosis	[83]
	OVCAR-3, PA-1, SKOV-3	10–100 μM		Inhibition of cell growth, cell cycle arrest, induction of apoptosis	[84]
	OVCAR-3	0–200 μM		Inhibition of cell proliferation and migration	[85]
	CAOV-3, OVCAR-3, SKOV3	5, 10, 20, 40, 80 µg/mL	BALB/c mice (4–5 weeks old); EGCG dose: 10, 30, 50 mg/kg	Inhibition of cell proliferation and induction of apoptosis; increased inhibition of tumor growth compared to paclitaxel (50 mg/kg EGCG vs. 5 mg/kg paclitaxel)	[86]
	SKOV-3	20, 30, 40, 50 µg/mL		Inhibition of cell proliferation and viability, DNA damage, induction of apoptosis	[87]
	A2780, SKOV3	0–20 nM	BALB/c mice; EGCG dose: 200 mg/kg, intratumoral injection	Inhibition of cell migration and survival, acceleration of apoptosis, inhibition of tumor growth	[88]
	HEY, OVCA 433	10, 20, 40 μM	Athymic (nu^+^/nu^+^) mice (4–6 weeks old); EGCG source: green tea leaves (12.4 g/L), drinking fluid	Inhibition of cell growth, induction of apoptosis, inhibition of angiogenesis and invasiveness; reduction in tumor growth	[89]
